# Supplementary material for: Surface Microbiome Profiling of Dental Elevators Using Third‐Generation Sequencing: Implications for Infection Control in Dental Practice
Source: Microbiologyopen. 2025 Dec 8;14(6):e70178. doi: 10.1002/mbo3.70178 (PMC12685768; doi:10.1002/mbo3.70178)
Supplement: Supplementary file 1 — customer_backup. [file MBO3-14-e70178-s001.zip › customer_backup/customer_backup/taxa_summary/heatmap/treat/treat.species.reabundance.heatmap.pdf]

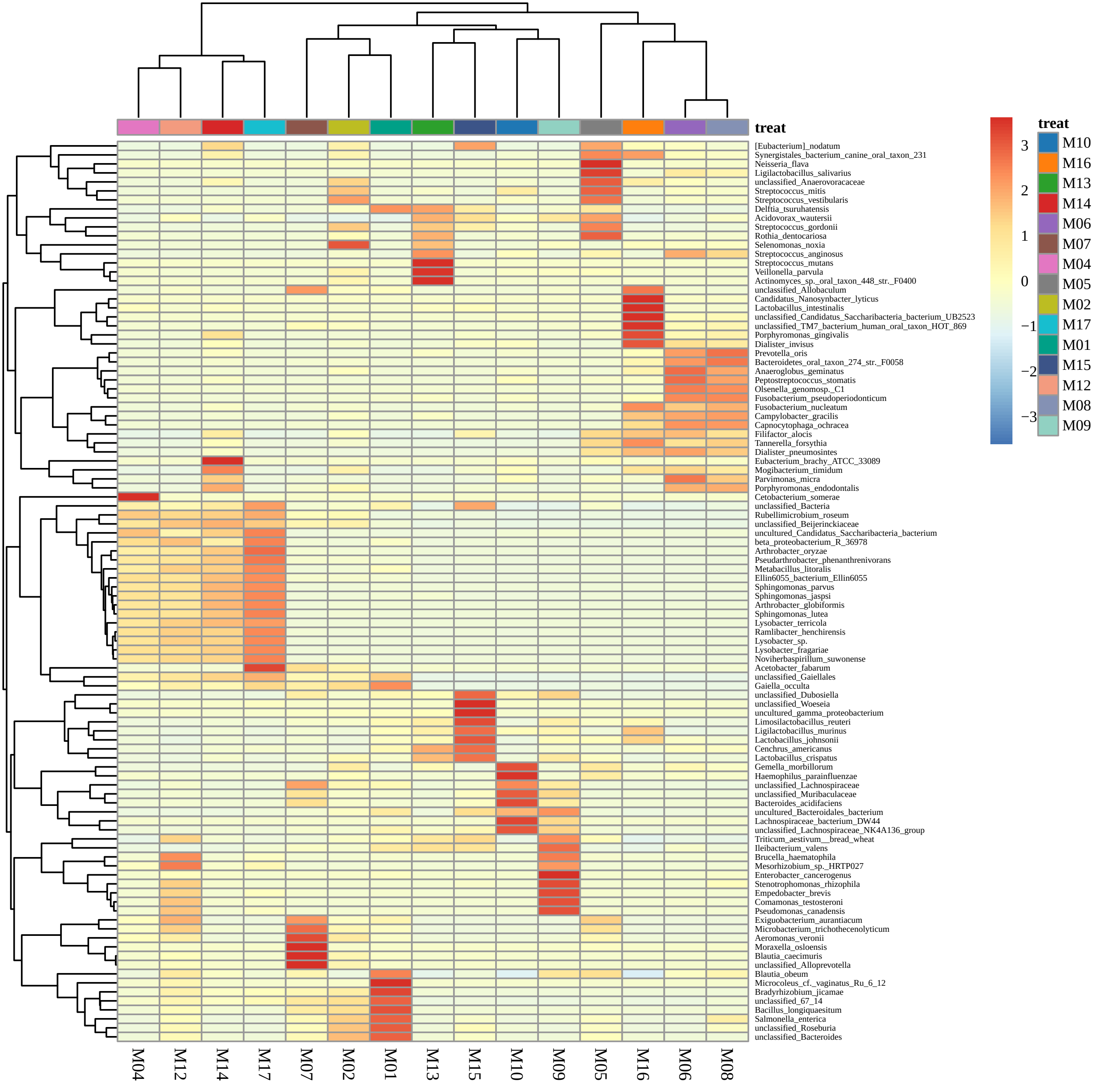
treat
[Eubacterium]_nodatum
Synergistales_bacterium_canine_oral_taxon_231
Neisseria_flava
Ligilactobacillus_salivarius
unclassified_Anaerovoracaceae
Streptococcus_mitis
Streptococcus_vestibularis
Delftia_tsuruhatensis
Acidovorax_wautersii
Streptococcus_gordonii
Rothia_dentocariosa
Selenomonas_noxia
Streptococcus_anginosus
Streptococcus_mutans
Veillonella_parvula
Actinomyces_sp._oral_taxon_448_str._F0400
unclassified_Allobaculum
Candidatus_Nanosynbacter_lyticus
Lactobacillus_intestinalis
unclassified_Candidatus_Saccharibacteria_bacterium_UB2523
unclassified_TM7_bacterium_human_oral_taxon_HOT_869
Porphyromonas_gingivalis
Dialister_invisus
Prevotella_oris
Bacteroidetes_oral_taxon_274_str._F0058
Anaeroglobus_geminatus
Peptostreptococcus_stomatis
Olsenella_genomosp._C1
Fusobacterium_pseudoperiodonticum
Fusobacterium_nucleatum
Campylobacter_gracilis
Capnocytophaga_ochracea
Filifactor_alocis
Tannerella_forsythia
Dialister_pneumosintes
Eubacterium_brachy_ATCC_33089
Mogibacterium_timidum
Parvimonas_micra
Porphyromonas_endodontalis
Cetobacterium_somerae
unclassified_Bacteria
Rubellimicrobium_roseum
unclassified_Beijerinckiaceae
uncultured_Candidatus_Saccharibacteria_bacterium
beta_proteobacterium_R_36978
Arthrobacter_oryzae
Pseudarthrobacter_phenanthrenivorans
Metabacillus_litoralis
Ellin6055_bacterium_Ellin6055
Sphingomonas_parvus
Sphingomonas_jaspsi
Arthrobacter_globiformis
Sphingomonas_lutea
Lysobacter_terricola
Ramlibacter_henchirensis
Lysobacter_sp.
Lysobacter_fragariae
Noviherbaspirillum_suwonense
Acetobacter_fabarum
unclassified_Gaiellales
Gaiella_occulta
unclassified_Dubosiella
unclassified_Woeseia
uncultured_gamma_proteobacterium
Limosilactobacillus_reuteri
Ligilactobacillus_murinus
Lactobacillus_johnsonii
Cenchrus_americanus
Lactobacillus_crispatus
Gemella_morbillorum
Haemophilus_parainfluenzae
unclassified_Lachnospiraceae
unclassified_Muribaculaceae
Bacteroides_acidifaciens
uncultured_Bacteroidales_bacterium
Lachnospiraceae_bacterium_DW44
unclassified_Lachnospiraceae_NK4A136_group
Triticum_aestivum__bread_wheat
Ileibacterium_valens
Brucella_haematophila
Mesorhizobium_sp._HRTP027
Enterobacter_cancerogenus
Stenotrophomonas_rhizophila
Empedobacter_brevis
Comamonas_testosteroni
Pseudomonas_canadensis
Exiguobacterium_aurantiacum
Microbacterium_trichothecenolyticum
Aeromonas_veronii
Moraxella_osloensis
Blautia_caecimuris
unclassified_Alloprevotella
Blautia_obeum
Microcoleus_cf._vaginatus_Ru_6_12
Bradyrhizobium_jicamae
unclassified_67_14
Bacillus_longiquaesitum
Salmonella_enterica
unclassified_Roseburia
unclassified_Bacteroides
M04
M12
M14
M17
M07
M02
M01
M13
M15
M10
M09
M05
M16
M06
M08
3
2
1
0
−1
−2
−3
treat
M10
M16
M13
M14
M06
M07
M04
M05
M02
M17
M01
M15
M12
M08
M09
